# Association of burnout and working environment conditions in respiratory care professionals in Saudi Arabia: a cross-sectional study

**DOI:** 10.3389/fpubh.2024.1434472

**Published:** 2024-12-05

**Authors:** Saad Al-Anazi, Syed Shahid Habib, Thamir Al-khlaiwi, Abdulaziz Alhomaidi Alodhayani, Abdulmueen Alotaibi, Saja Aldulejan, Sufana Al Safadi, Fahad Saad Alshammari, Aqeelah Marar, Afaf Alrashdi, Alhanouf G. Almutairi, Mohammed Alshahrani

**Affiliations:** ^1^Department of Physiology, College of Medicine, King Saud University, Riyadh, Saudi Arabia; ^2^Azeer Medical Company, Riyadh, Saudi Arabia; ^3^Health Promotion and Health Education Research Chair, Medicine College, King Saud University, Riyadh, Saudi Arabia; ^4^Department of Anaesthesia Technology, College of Applied Sciences, University of Almaarefa, Dariyah, Saudi Arabia; ^5^Education Department, Respiratory Care Administration, Riyadh Second Heath Cluster, King Fahad Medical City, Riyadh, Saudi Arabia; ^6^Department of Respiratory Care, College of Applied Medical Science, Imam Abdulrahman Bin Faisal University, Damam, Saudi Arabia; ^7^Ministry of Health - Hospital Administration Affairs, Commissioning Hospital Department, Diriyah Hospital, Riyadh, Saudi Arabia; ^8^Department of Respiratory Critical Care, Respiratory Care Administration, Riyadh Second Heath Cluster, King Fahad Medical City, Riyadh, Saudi Arabia; ^9^Department of Rehabilitation Sciences, College of Health and Rehabilitation Sciences, Princess Nourah Bint Abdulrahman University, Riyadh, Saudi Arabia; ^10^Department of Respiratory Services, King Abdulaziz Medical City, Riyadh, Saudi Arabia

**Keywords:** burnout, respiratory therapists, sociodemographic factors, professional factors, personal achievement, multinomial logistic regression

## Abstract

**Introduction:**

Burnout is a pervasive issue in healthcare, and it impacts both the wellbeing of healthcare professionals and the quality of patient care. This study examines factors associated with burnout and working environment among respiratory therapists in Saudi Arabia.

**Methods:**

A structured questionnaire was used to collect data from respiratory therapists. We collected 315 questionnaires including burnout assessment. Multinomial logistic regression was used to assess the relationship between various variables and the likelihood of experiencing severe burnout and personal achievement problems.

**Results:**

Marital status was a significant predictor of severe burnout (*p* = 0.001), with single individuals having 8.2 times higher odds than married individuals. Working longer than 44 h per week was associated with a 19.3-fold increase in the odds of severe personal achievement issues compared to those working 40–44 h per week (*p* < 0.001). Age, gender, living arrangements, education, employment status, and salary level were not significant predictors of severe burnout or personal achievement issues.

**Conclusion with discussion:**

The findings highlight that marital status and extended working hours are significantly associated with increased burnout among respiratory therapists in Saudi Arabia. These results underscore the importance of social support networks and work-life balance in mitigating burnout risks within this profession.

## Introduction

Burnout is a widespread and significant issue with profound consequences for both individuals and organizations. It is characterized by three primary dimensions: emotional exhaustion, depersonalization, and reduced personal accomplishment (1, 2). Recent research indicates that burnout affects a considerable proportion of respiratory care professionals (RCPs), with a 2021 study revealing a 72% prevalence rate (3). Studies conducted in Kuwait and Saudi Arabia shed light on the prevalence of burnout among RCPs in the region. In Kuwait, 40% of RCPs were found to exhibit high levels of burnout, with emotional exhaustion emerging as the most prevalent symptom (4). Similarly, a study in Saudi Arabia reported that 42.4% of RCPs experienced significant burnout, with factors like protracted working hours, heavy workloads, and a lack of organizational support identified as contributing factors (5).

Burnout among RCPs is not limited to the Middle East. Studies from various regions around the world have reported similar findings. For example, a study in the United States found a prevalence of burnout among RCPs ranging from 25 to 75% (6). In Europe, a meta-analysis revealed that 30% of RCPs experienced high levels of burnout (7).

These studies highlight the global nature of burnout among RCPs, suggesting that this is a widespread issue that affects healthcare professionals in various cultural and socioeconomic contexts.

The main contributors to burnout epidemic include factors such as inadequate leadership, excessive workloads, and understaffing (3). RCPs shoulder a myriad of job demands, including extended working hours, exposure to infectious agents, and the emotional strain of caring for critically ill patients (1, 2). These demanding work conditions place RCPs at a heightened risk of experiencing burnout.

The repercussions of burnout are far-reaching and extend to both individual wellbeing and organizational performance. Burnout has been linked to various adverse outcomes, encompassing reduced productivity, heightened absenteeism, elevated turnover rates, compromised quality of care provided by respiratory professionals, and unfavorable patient consequences (8).

The consequences of burnout encompass both physical and mental health issues. Physical health repercussions can manifest as fatigue, headaches, gastrointestinal problems, and an increased risk of heart disease (1). On the mental health front, burnout may lead to anxiety, depression, and substance abuse (9), exacerbating interpersonal conflicts and diminishing self-esteem (1). Organizations also bear the brunt of burnout, facing diminished productivity, higher absenteeism, and increased turnover rates (1). Moreover, compromised care quality provided by burnt-out respiratory professionals can culminate in adverse patient outcomes (25). Additionally, gender and years of experience have surfaced as factors associated with burnout among respiratory therapists in Saudi Arabia, with female respiratory therapists and those with <5 years of experience being more susceptible to burnout (8).

Efforts to prevent burnout among RCPs should encompass measures such as ensuring adequate staffing levels, reducing workloads, fostering positive work environments, and offering opportunities for professional growth (1). Recognizing the signs and symptoms of burnout and seeking assistance when needed are pivotal. Multiple resources are available to aid RCPs grappling with burnout, including counseling, support groups, and stress management programs (1).

While several studies have explored burnout prevalence among healthcare professionals in Saudi Arabia, including RCPs, further comprehensive investigation of various factors affecting respiratory therapists is warranted to delineate the specific prevalence of burnout across RCPs in Saudi Arabia. This in-depth understanding of burnout's prevalence across diverse roles can provide valuable insights into the contributing factors and pave the way for targeted interventions aimed at mitigating its impact. Hence, this study endeavors to assess variables associated with burnout prevalence among RCPs in Saudi Arabia through a cross-sectional questionnaire design. By scrutinizing the variations in burnout rates and identifying associated factors, this research will enrich the knowledge on burnout among RCPs and facilitate the development of effective strategies to prevent or manage burnout within this distinct healthcare context.

## Methodology

In this cross-sectional questionnaire study conducted from January 1 to February 29, 2023, we investigated the pervasive issue of burnout among RCPs in Saudi Arabia. We aimed to comprehensively evaluate burnout variables within the respiratory care sector and identify associated factors that contribute to this phenomenon. The methodology employed for this study adhered to stringent ethical guidelines, ensuring participant confidentiality and research integrity.

### Sample selection

For our study, we carefully collected a sample of 315 RCPs from diverse healthcare facilities in Saudi Arabia. We employed a stratified random sampling method to select participants from a diverse pool of RCPs in Saudi Arabia. The population was stratified based on work setting (hospitals, clinics, long-term care facilities) and job position (respiratory technicians, respiratory therapists, supervisors, heads and above). A random sample of RCPs was then selected from each stratum to ensure representation of different groups.

#### Inclusion criteria

- RCPs currently employed in Saudi Arabia.
- Those working in different positions within the field, such as respiratory technicians, respiratory therapists, respiratory therapy supervisors, and respiratory therapy heads and above.
- Individuals employed in various healthcare facilities, including hospitals, clinics, and long-term care facilities.
- Willingness to participate in the questionnaire study.

#### Exclusion criteria

- Non-RCPs.
- Participants are unwilling to complete the questionnaire.

### Data collection

Data were collected using a self-administered questionnaire that incorporated validated scales, including the Maslach Burnout Inventory (MBI-HSS MP). Additionally, the questionnaire gathered demographic and work-related variables. The survey was distributed electronically to the selected cohort via Google Forms to facilitate ease of access and completion.

### Measuring burnout

Maslach Burnout Inventory for Medical Personnel (MBI-HSS(MP) (10). Was our primary tool for assessing burnout levels among RCPs in various positions. This inventory, which consists of three domains—personal accomplishment (eight items), depersonalization (five items), and emotional exhaustion (nine items)—provided a comprehensive insight into the respondents' burnout experiences. We received responses from all 315 RCPs. We also collected data on the respondents' sex, age, marital status, shift timing, years of experience, and their work settings, whether critical or general care.

### Scoring for burnout levels

To categorize burnout levels, we calculated scale scores based on the responses to the inventory items within each domain. High scores in emotional exhaustion and depersonalization, along with low scores in personal accomplishment, were indicative of high burnout levels. Following Maslach and Jackson's criteria (11), a score of 27 for emotional exhaustion, 10 for depersonalization, and 33 for personal achievement defined high-level burnout. Moderate burnout was denoted by scores of 19–26 for emotional exhaustion, 6–9 for depersonalization, and 34–39 for personal achievement. Low burnout levels were defined by scores of 18 for emotional exhaustion, five for depersonalization, and 40 for personal achievement. We also collected data on the respondents' respiratory therapy positions and income status.

### Ethical considerations

To ensure the ethical integrity of our study, we obtained prior approval from the ALMaarefa University Institutional Review Board (IRB 23-010). Participants provided informed consent, were assured of confidentiality and data protection, and were informed of their right to withdraw from the study at any time. Data was anonymized by removing personal identifiers to protect participant privacy.

### Data analysis

Our data analysis approach employed quantitative methods to examine burnout levels across various RCP positions. Descriptive statistics, including means, frequencies, and percentages, were utilized to provide a comprehensive overview of our sample.

- **Identifying factors contributing to burnout**: We applied **multinomial logistic regression**, which is well-suited for modeling categorical outcomes such as burnout levels (low, moderate, severe). The assumption of independence among categories, known as the **independence of irrelevant alternatives (IIA)**, implies that the characteristics of one particular choice alternative do not affect the relative probabilities of choosing the other alternatives. This assumption is well-documented and supported by both theoretical and empirical research (12–14).
- **Statistical significance**: To ensure the robustness of our findings, we set the threshold for statistical significance at ***p*** **≤ 0.05**, aligning with established research practices.

## Results

The total sample size comprised 315 respiratory therapists. According to Table 1, the majority of the participants were male (53.3%, *n* = 168) and Saudi nationals (92.1%, *n* = 290). The participants were relatively young, with the majority (38.4%) falling within the age range of 26–30 years. Most participants held an undergraduate degree (73.0%) and worked 40–44 h per week (32.1%). Additionally, 66.3% were respiratory therapists, and 73.3% worked in critical care areas. Furthermore, most participants had a patient-workload ratio of 1–10 (26.0%) (Table 2).

**Table 1:** Sociodemographic characteristics of the subjects (*N* = 315).

**Variables**		***N* =315**	**%**
Age	Below 25	73	23.2
	26–30	121	38.4
	31–40	93	29.5
	Above 40	28	8.9
Gender	Male	168	53.3
	Female	147	46.7
Nationality	Saudi	290	92.1
	Non-Saudi	25	7.9
Live with family	Yes	84	26.7
	No	231	73.6
Level of education	Diploma	16	5.1
	Undergraduate (bachelor's degree)	230	73
	Postgraduate (Master, PhD)	69	21.9
Employment status	Governmental sector employee	250	79.4
	Private sector employee	65	20.6
Marital status	Single	171	54.3
	Married	139	44.1
	Divorced	5	1.6
Salary status	10,000–15,000 SAR	168	53.3
	15,000–20,000 SAR	35	11.1
	Above 20,000 SAR	24	7.6
	Below 10,000 SAR	88	27.9
Job experience	1–5 years	180	57.1
	5–10 years	69	21.9
	Above 10 years	66	21
Work position	Head	38	12.1
	Respiratory technician	28	8.9
	Respiratory therapist 1	209	66.3
	Supervisor	40	12.7
Working hours	40–44 h	101	32.1
	Above 44 h	199	63.2
	Below 40 h	15	4.8
Working location	Critical area	231	73.3
	Non-critical area	84	26.7
Work duty	Change duty (day and night)	147	46.7
	Day shift	138	43.8
	Night shift	30	9.5
Workload	1–10 ratio	82	26
	1–4 ratio	21	6.7
	1–6 ratio	79	25.1
	Above 1–10 ratio	76	24.1
	Not required (office work)	57	18.1

**Table 2:** Association of sociodemographic characteristics with burnout, depersonalization, personal achievement in respiratory therapists (*N* = 315).

**Section**	**Category**	**Male**		**Female**		***p*-value**
A: Burnout	Low	(58) 59.8%		(39) 40.2%		0.169
	Moderate	(56) 54.4%		(47) 45.6%		
	Severe	(54) 47.0%		(61) 53.0%		
B: Depersonalization	Low	(28) 52.8%		(25) 47.2%		0.699
	Moderate	(37) 49.3%		(38) 50.7%		
	Severe	(103) 55.1%		(84) 44.9%		
C: Personal achievement	Low	(41) 46.1%		(48) 53.9%		0.266
	Moderate	(46) 56.8%		(35) 43.2%		
	Severe	(81) 55.9%		(64) 44.1%		
**Section**	**Category**	**Saudi**		**Non-Saudi**		***p*** **-value**
A: Burnout	Low	(86) 88.7%		(11) 11.3%		0.258
	Moderate	(95) 92.2%		(8) 7.8%		
	Severe	(109) 94.8%		(6) 5.2%		
B: Depersonalization	Low	(43) 81.1%		(10) 18.9%		0.005^*^
	Moderate	(70) 93.3%		(5) 6.7%		
	Severe	(177) 94.7%		(10) 5.3%		
C: Personal achievement	Low	(77) 86.5%		(12) 13.5%		0.002^*^
	Moderate	(71) 87.7%		(10) 12.3%		
	Severe	(142) 97.9%		(3) 2.1%		
**Section**	**Category**	**Live with family**		**Non-live with family**		***p*** **-value**
A: Burnout	Low	(78) 80.4%		(19) 19.6%		0.045^*^
	Moderate	(67) 65.0%		(36) 35.0%		
	Severe	(86) 74.8%		(29) 25.2%		
B: Depersonalization	Low	(41) 77.4%		(12) 22.6%		0.585
	Moderate	(52) 69.3%		(23) 30.7%		
	Severe	(138) 73.8%		(49) 26.2%		
C: Personal achievement	Low	(60) 67.4%		(29) 32.6%		0.321
	Moderate	(62) 76.5%		(23) 30.7%		
	Severe	(109) 75.2%		(36) 24.8%		
**Section**	**Category**	**Diploma**	**Undergraduate**	**Postgraduate**		***p*** **-value**
A: Burnout	Low	(0) 9.3%	(61) 62.9%	(27) 27.8%		0.037^*^
	Moderate	(5) 4.9%	(78) 75.7%	(20) 19.4%		
	Severe	(2) 1.7%				
B: Depersonalization	Low	(7) 13.2%	(31) 58.5%	(15) 28.3%		0.003^*^
	Moderate	(6) 8.0%	(56) 74.7%	(13) 17.3%		
	Severe	(3) 1.6%				
C: Personal achievement	Low	(6) 6.7%	(60) 67.4%	(23) 25.8%		0.125
	Moderate	(5) 6.2%	(66) 81.5%	(10) 12.3%		
	Severe	(5) 3.4%	(104) 71.7%	(36) 24.8%		
**Section**	**Category**	**Single**	**Married**	**Divorced**		***P*** **-value**
A: Burnout	Low	(47) 48.5%	(50) 51.5%	(0) 0.0%		0.153
	Moderate	(54) 52.4%	(46) 44.7%	(3) 2.9%		
	Severe	(70) 60.9%	(43) 37.4%	(2) 1.7%		
B: Depersonalization	Low	(23) 43.4%	(29) 54.7%	(1) 1.9%		0.022^*^
	Moderate	(32) 42.7%	(41) 54.7%	(2) 2.7%		
	Severe	(116) 62.0%	(69) 36.9%	(2) 1.1%		
C: Personal achievement	Low	(49) 55.1%	(38) 42.7%	(2) 2.2%		0.684
	Moderate	(40) 49.4%	(39) 48.1%	(2) 2.5%		
	Severe	(82) 56.6%	(62) 42.8%	(1) 0.7%		
**Section**	**Category**	**Below 25**	**26–30**	**31–40**	**Above 40**	***p*** **-value**
A: Burnout	Low	(25) 25.8%	(29) 29.9%	(31) 32.0%	(12) 12.4%	0.221
	Moderate	(21) 20.4%	(41) 39.8%	(30) 29.1%	(11) 10.7%	
	Severe	(27) 23.5%	(51) 44.3%	(32) 27.8%	(5) 4.3%	
B: Depersonalization	Low	(10) 18.9%	(16) 30.2%	(18) 34.0%	(9) 17.0%	0.174
	Moderate	(19) 25.3%	(25) 33.3%	(24) 32.0%	(7) 9.3%	
	Severe	(44) 23.5%	(80) 42.8%	(51) 27.3%	(12) 6.4%	
C: Personal achievement	Low	(20) 22.5%	(28) 31.5%	(30) 33.7%	(11) 12.4%	0.115
	Moderate	(18) 22.2%	(29) 35.8%	(23) 28.4%	(11 13.6%	
	Severe	(35) 24.1%	(64) 44.1%	(40) 27.6%	(6) 4.1%	

* Significant. Data is presented as number and percent.

**Figure 1:**
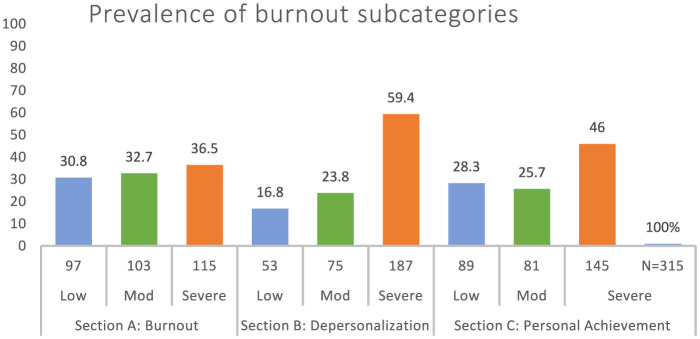
Prevalence of burnout subcategories.

Figure 1 illustrates the distribution of burnout levels among participants: 36.5% (*n* = 115) experienced severe burnout, 32.7% had moderate burnout, and 30.8% had low burnout levels. Regarding the subdomains of burnout, 59.4% of participants exhibited severe depersonalization, followed by 23.8% with moderate depersonalization. In contrast, 46.0% of participants reported severe personal accomplishment issues, followed by 25.7% with moderate personal accomplishment issues.

Table 3 presents the correlation between burnout and work experience. Participants with more than 10 years of experience were more likely to experience burnout than those with <5 years of experience (*p* = 0.052), indicating a marginally significant association. Table 4 shows the correlation between burnout and job title. Supervisors were more likely to experience burnout than respiratory therapists or respiratory technicians (*p* = 0.067), which approaches statistical significance.

**Table 3:** Association of burnout, depersonalization, personal achievement, and work environment of respiratory therapist (*N* = 315).

**Section**	**Category**		**Critical (%)**		**Noncritical (%)**		***p*-value**
A: Burnout	Low		(67) 69.1%		(30) 30.9%		0.516
	Moderate		(77) 78.8%		(25) 25.2%		
	Severe		(87) 75.7%		(28) 24.3%		
B: Depersonalization	Low		(36) 67.9%		(17) 32.1%		0.619
	Moderate		(56) 74.7%		(19) 25.3%		
	Severe		(139) 74.3%		(48) 25.7%		
C: Personal achievement	Low		(61) 68.5%		(28) 31.5%		0.303
	Moderate		(64) 79.0%		(17) 21.0%		
	Severe		(106) 73.1%		(39) 26.9%		
**Section**	**Category**		**Governmental (%)**		**Private** ***n*** **(%)**		***p*** **-value**
A: Burnout	Low		(77) 79.4%		(20) 20.6%		0.97
	Moderate		(81) 78.6%		(22) 21.4%		
	Severe		(92) 80.0%		(23) 20.0%		
B: Depersonalization	Low		(40) 75.5%		(13) 24.5%		0.744
	Moderate		(60) 80.0%		(15) 20.0%		
	Severe		(150) 80.2%		(37) 19.8%		
C: Personal achievement	Low		(65) 73.0%		(24) 27.0%		0.198
	Moderate		(65) 80.2%		(16) 19.8%		
	Severe		(120) 82.8%		(25) 17.2%		
**Section**	**Category**		**Below 40 h**	**40–44**	**Above 44 h**		***p*** **-value**
A: Burnout	Low		(54) 55.7%	(2) 1.9%	(1) 9%		0.061
	Moderate		(31) 32.0%	(38) 36.9%	(32) 27.8%		
	Severe		(12) 12.4%	(63) 61.2%	(82) 71.3%		
B: Depersonalization	Low		(33) 62.3%	(7) 9.3%	(0) 0%		0.021^*^
	Moderate		(15) 28.3%	(27) 36.0%	(59) 31.6%		
	Severe		(5) 9.4%	(41) 54.7%	(125) 66.8%		
C: Personal achievement	Low		(56) 62.9%	(25) 28.1%	(8) 9.0%		0.08
	Moderate		(0) 0%	(26) 32.1%	(55) 67.9%		
	Severe		(0) 0%	(50) 34.5%	(88) 60.7%		
**Section**	**Category**		**Day shift**	**Night Shift**	**Change duty**		***p*** **-value**
A: Burnout	Low		(45) 46.4%	(7) 7.2%	(45) 46.4%		0.251
	Moderate		(51) 49.5%	(8) 7.8%	(44) 42.7%		
	Severe		(42) 36.5%	(15) 13.0%	(58) 50.4%		
B: Depersonalization	Low		(33) 62.3%	(4) 7.5%	(16) 30.2%		0.006^*^
	Moderate		(24) 32.0%	(5) 6.7%	(46) 61.3%		
	Severe		(81) 43.3%	(21) 11.2%	(85) 45.5%		
C: Personal achievement	Low		(45) 50.6%	(10) 11.2%	(34) 38.2%		0.386
	Moderate		(36) 44.4%	(7) 8.6%	(38) 46.9%		
	Severe		(57) 39.3%	(13) 9.0%	(75) 51.7%		
**Section**	**Category**		**1–5 years**	**5–10 years**	**Above 10 years**		***p*** **-value**
A: Burnout	Low		(50) 51.5%	(21) 21.6%	(26) 26.8%		0.202
	Moderate		(58) 56.3%	(21) 20.4%	(24) 23.3%		
	Severe		(72) 62.6%	(27) 23.5%	(16) 13.9%		
B: Depersonalization	Low		(12) 22.6%	(18) 34.0%	(23) 43.4%		0.052^*^
	Moderate		(16) 21.3%	(18) 24.0%	(41) 54.7%		
	Severe		(41) 21.9%	(30) 16.0%	(116) 62.0%		
C: Personal achievement	Low		(19) 21.3%	(23) 25.8%	(47) 52.8%		0.12
	Moderate		(18) 22.2%	(22) 27.2%	(41) 50.6%		
	Severe		(32) 22.1%	(21) 14.5%	(92) 63.4%		
**Section**	**Category**		**500–1,000 beds**	**Above 1,000**	<**500 beds**		***p*** **-value**
				**bed**			
A: Burnout	Low		(29) 29.9%	(19) 19.6%	(49) 50.5%		0.172
	Moderate		(30) 29.1%	(25) 24.3%	(48) 46.6%		
	Severe		(45) 39.1%	(30) 26.1%	(40) 34.8%		
B: Depersonalization	Low		(12) 22.6%	(14) 26.4%	(27) 50.9%		0.281
	Moderate		(27) 36.0%	(25) 24.3%	(35) 46.7%		
	Severe		(45) 39.1%	(30) 26.1%	(75) 40.1%		
C: Personal achievement	Low		(27) 30.3%	(25) 28.1%	(37) 41.6%		0.769
	Moderate		(28) 34.6%	(19) 23.5%	(34) 42.0%		
	Severe		(49) 33.8%	(30) 20.7%	(66) 45.5%		
**Section**	**Category**	**Below 10,000**	**10,000–15,000**	**15,000–20,000**	**Above 20,000**		***p*** **-value**
A: Burnout	Low	(25) 25.8%	(16) 16.5%	(11) 11.3%	(45) 46.4%		0.09
	Moderate	(27) 26.2%	(13) 12.6%	(6) 5.8%	(57) 55.3%		
	Severe	(36) 31.3%	(6) 5.2%	(7) 6.1%	(66) 57.4%		
B: Depersonalization	Low	(14) 26.4%	(14) 26.4%	(3) 5.7%	(22) 41.5%		0.004^*^
	Moderate	(20) 26.7%	(10) 13.3%	(7) 9.3%	(38) 50.7%		
	Severe	(54) 28.9%	(11) 5.9%	(14) 7.5%	(108) 57.8%		
C: Personal achievement	Low	(28) 31.5%	(16) 18.0%	(6) 6.7%	(39) 43.8%		0.087
	Moderate	(20) 24.7%	(9) 11.1	(9) 11.1%	(43) 53.1%		
	Severe	(40) 27.6%	(10) 6.9%	(9) 6.2%	(86) 59.3%		
**Section**	**Category**	**Head**	**Respiratory technician**	**Respiratory therapist 1**	**Supervisor**		***p*** **-value**
A: Burnout	Low	(18) 18.6%	(10) 10.3%	(61) 62.9%	(8) 8.2%		0.102
	Moderate	(12) 11.7%	(8) 7.8%	(65) 63.1%	(18) 17.5%		
	Severe	(8) 7.0%	(10) 8.7%	(83) 72.2%	(14) 12.2%		
B: Depersonalization	Low	(12) 22.6%	(4) 7.5%	(30) 56.6%	(7) 13.2%		0.182
	Moderate	(8) 10.7%	(9) 12.0%	(51) 68.0%	(7) 9.3%		
	Severe	(18) 9.6%	(15) 8.0%	(128) 68.4%	(26) 13.9%		
C: Personal achievement	Low	(13) 14.6%	(11) 12.4 %	(48) 53%	(17) 19.1 %		0.1117
	Moderate	(11) 13.6%	(6) 7.47%	(55) 67.6%	(9) 11.1%		
	Severe	(14) 9.7%	(11) 7.6%	(106) 73.1%	(14) 9.7%		
**Section**	**Category**	**1–10**	**1–4**	**1–6**	**Above 1–10**	**Office work**	***p*** **-value**
A: Burnout	Low	(23) 23.7%	(11) 11.3%	(25) 25.8%	(16) 16.5%	(22) 22.7%	0.082
	Moderate	(27) 26.2%	(6) 5.8%	(27) 26.2%	(23) 22.3%	(20) 19.4%	
	Severe	(32) 27.8%	(4) 3.5%	(27) 23.5%	(37) 32.2%	(15) 13.0%	
B: Depersonalization	Low	(13) 24.5%	(6) 11.3%	(12) 22.6%	(8) 15.1%	(14) 26.4%	0.184
	Moderate	(18) 24.0%	(7) 9.3%	(22) 29.3%	(15) 20.0%	(13) 17.3%	
	Severe	(51) 27.3%	(8) 4.3%	(45) 24.1%	(53) 28.3%	(30) 16.0%	
C: Personal achievement	Low	(25) 28.1%	(7) 7.9%	(16) 18.0%	(20) 22.5%	(21) 23.6%	0.364
	Moderate	(24) 29.6%	(3) 3.7%	(19) 23.5%	(20) 24.7%	(15) 18.5%	
	Severe	(33) 22.8%	(11) 7.6%	(44) 30.0%	(36) 24.8%	(21) 14.5%	

* Significant. Data is presented as number and percent.

**Table 4:** Association of variables with severe burnouts (*N*=315).

**Variable**	**Subcategory**	***N***	**OR**	**95% CI**	***p*-value**
Age	Below 25	73	0.44	0.044–4.404	0.000^*^
	26–30	121	1.356	0.165–11.132	
	31–40	93	1.756	0.284–10.859	
	Above 40	28	1	–	
Gender	Male	168	0.561	0.277–1.138	0.109
	Female	147	1	–	
Living with family	No	231	1.318	0.571–3.044	0.518
	Yes	84	1	–	
Level of Education	Diploma	16	0.221	0.032–1.505	0.123
	(Bachelor's degree)	230	1.173	0.492–2.798	
	(Master, PhD)	69	1	–	
Employment status	Governmental	250	1.287	0.508–3.260	0.594
	Private	65	1	–	
Marital Status	Single	171	8.2736	3.763–1.818	0.000^*^
	Married	139	5.367	5.367–5.367	
	Divorced	5	1	–	
Salary Status	10,000–15,000 SAR	168	0.566	0.212–1.513	0.256
	15,000–20,000 SAR	35	0.19	0.042-.867	
	Above 20,000 SAR	24	0.668	0.095–4.719	
	Below 10,000 SAR	88	1	–	
Job Experience	1–5 years	180	0.838	0.163–4.306	0.755
	5–10 years	69	0.813	0.222–2.980	
	Above 10 years	66	1	–	
Work position	Head	38	0.261	0.062–1.099	0.067
	Respiratory technician	28	0.402	0.071–2.287	
	Respiratory Therapist 1	209	0.402	0.107–1.516	
	Supervisor	40	1	–	
Working hours per week	40–44 h	101	14.185	1.523–132.094	0.02^*^
	Above 44 h	199	19.351	2.150–174.196	
	Below 40 h	15	1	–	
Working location inside the hospital	Critical area	231	1.153	0.430–3.091	0.777
	Non-critical area	84	1	–	
Shift duty	Change duty (day and night)	147	0.458	0.133–1.573	0.215
	Day shift	138	0.511	0.139–1.883	
	Night shift	30	1	–	
Workload patient ratio per shift	1–10 ratio	82	2.834	0.661–12.161	0.161
	1–4 ratio	21	1.703	0.287–10.098	
	1–6 ratio	79	2.319	0.565–9.525	
	Above 1–10 ratio	76	4.513	1.115–18.270	
	(Office work)	57	1	–	

* Significant.

Results from multinomial logistic regression.

Additionally, based on a *p*-value of 0.123, there was no statistically significant difference in burnout levels between respiratory therapists with postgraduate degrees and those with diploma or undergraduate degrees (Table 4).

Table 5 presents the association of variables with burnout using multinomial logistic regression analysis. Age between 31 and 40 years was associated with a 1.7 times higher likelihood of severe burnout (*p* < 0.001). Participants aged 26–30 years had a 1.3 times higher likelihood of severe burnout (*p* = 0.045). Marital status was also associated with severe burnout, with single participants having an 8.2 times higher likelihood compared to married participants (*p* < 0.001). Working hours per week showed a significant association with severe burnout: participants working above 44 h per week had a 19.3 times higher likelihood compared to those working 40–44 h per week (*p* < 0.001).

**Table 5:** Association of variables with severe depersonalization (*N* = 315).

**Variable**	**Subcategory**	***N***	**OR**	**95% CI**	***P*-value**
Age	Below 25	73	0.33	0.026–4.207	0.16
	26–30	121	0.557	0.059–5.279	
	31–40	93	0.68	0.104–4.462	
	Above 40	28	1	–	
Gender	Male	168	1.877	0.810–4.348	0.142
	Female	147	1	–	
Living with family	No	231	2.205	0.748–6.496	0.152
	Yes	84	1	–	
Level of education	Diploma	16	0.062	0.009-.440	0.005^*^
	(Bachelor's degree)	230	0.979	0.385–2.485	
	(Master, PhD)	69	1	–	
Employment status	Governmental	250	1.1	0.405–2.989	0.851
	Private	65	1	–	
Marital status	Single	171	2.543	0.148–43.808	0.52
	Married	139	0.751	0.045–12.504	
	Divorced	5	1	–	
Salary status	10,000–15,000 SAR	168	1.178	0.362–3.829	0.786
	15,000–20,000 SAR	35	0.236	0.048–1.151	
	Above 20,000 SAR	24	5.447	0.548–54.111	
	Below 10,000 SAR	88	1	–	
Job experience	1–5 years	180	1.49	0.217–10.219	0.685
	5–10 years	69	1.318	0.311–5.589	
	Above 10 years	66	1	–	
Work position	Head	38	0.214	0.051-.904	0.036^*^
	Respiratory technician	28	2.922	0.328–26.005	
	Respiratory therapist 1	209	0.464	0.112–1.927	
	Supervisor	40	1	–	
Working hours	40–44 h	101	8.252	1.339–50.847	0.023^*^
	Above 44 h	199	4.967	0.875–28.189	
	Below 40 h	15	1	–	
Working location	Critical area	231	1.266	0.402–3.986	0.686
	Non-critical area	84	1	–	
Shift duty	Change duty (day and night)	147	0.535	0.106–2.696	0.449
	Day shift	138	0.329	0.064–1.685	
	Night shift	30	1	–	
Workload patient ratio per shift	1–10 ratio	82	1.509	0.307–7.420	0.612
	1–4 ratio	21	0.86	0.135–5.456	
	1–6 ratio	79	1.556	0.317–7.636	
	Above 1–10 ratio	76	3.885	0.792–19.055	
	(Office work)	57	1	–	

* Significant.

Results from multinomial logistic regression.

Workload per shift also demonstrated an association with severe burnout. Participants with a patient-workload ratio of above 1–10 had a 2.8 times higher likelihood compared to those with a ratio of 1–4 (*p* = 0.002).

The odds of severe depersonalization were significantly higher for participants earning above 20,000 SAR (OR = 5.447, *p* = 0.023), respiratory therapists (OR = 0.464, *p* = 0.036), and participants working 40–44 h per week (OR = 8.252, *p* = 0.023) or above 44 h per week (OR = 4.967, *p* = 0.025) (Table 6). Notably, an OR <1 (e.g., OR = 0.464 for respiratory therapists) indicates a lower likelihood of severe depersonalization compared to the reference group.

**Table 6:** Association of variables with severe personal achievement (*N* = 315).

**Variable**	**Subcategory**	***N***	**OR**	**95% CI**	***p*-value**
Age	Below 25	73	1.232	0.139–10.944	0.994
	26–30	121	1.916	0.248–14.808	
	31–40	93	0.709	0.119–4.222	
	Above 40	28	1	–	
Gender	Male	168	1.751	0.885–3.466	0.142
	Female	147	1	–	
Living with family	No	231	0.813	0.396–1.669	0.573
	Yes	84	1	–	
Level of education	Diploma	16	0.823	0.140–4.817	0.829
	(Bachelor's degree)	230	0.694	0.305–1.580	
	(Master, PhD)	69	1	–	
Employment status	Governmental	250	1.397	0.609–3.201	0.43
	Private	65	1	–	
Marital status	Single	171	4.998	0.340–73.496	0.241
	Married	139	6.349	0.431–93.602	
	Divorced	5	1	–	
Salary status	10,000–15,000 SAR	168	1.153	0.473–2.809	0.754
	15,000–20,000 SAR	35	0.581	0.150–2.250	
	Above 20,000 SAR	24	1.688	0.263–10.842	
	Below 10,000 SAR	88	1	–	
Job experience	1–5 years	180	0.412	0.084–2.023	0.275
	5–10 years	69	0.689	0.200–2.375	
	Above 10 years	66	1	–	
Work position	Head	38	1.05	0.313–3.522	0.937
	Respiratory technician	28	2.956	0.655–13.334	
	Respiratory therapist 1	209	3.263	1.090–9.774	
	Supervisor	40	1	–	
Working hours per week	40–44 h	101	3.722	0.977–14.169	0.054^*^
	Above 44 h	199	2.242	0.628–8.003	
	Below 40 h	15	1	–	
Working location	Critical area	231	0.975	0.394–2.412	0.956
	Non-critical area	84	1	–	
Shift duty	Change duty (day and night)	147	1.28	0.416–3.942	0.667
	Day shift	138	1.182	0.364–3.834	
	Night shift	30	1	–	
Workload patient ratio per shift	1–10 ratio	82	0.866	0.229–3.282	0.832
	1–4 ratio	21	1.181	0.235–5.940	
	1–6 ratio	79	1.818	0.474–6.975	
	Above 1–10 ratio	76	1.332	0.376–4.715	
	(Office work)	57	1	–	

* Significant.

Results from multinomial logistic regression.

Moreover, participants with a patient-workload ratio above 1–10 had a 1.5 times higher likelihood of severe depersonalization (*p* = 0.002) (Table 6).

Regarding personal accomplishment issues, the odds of severe personal accomplishment were marginally higher for participants earning above 20,000 SAR (OR = 1.688, *p* = 0.054) and respiratory therapists (OR = 3.263, *p* = 0.054). However, the associations for male participants (OR = 1.751, *p* = 0.142), single participants (OR = 4.998, *p* = 0.241), and married participants (OR = 6.349, *p* = 0.241) were not statistically significant (Table 7).

**Table 7:** Association of variables with severe personal achievement (*N* = 315).

**Variable**	**Subcategory**	***N***	**OR**	**95% CI**	***p*-value**
Age	Below 25	73	1.232	0.139–10.944	0.994
	26–30	121	1.916	0.248–14.808	
	31–40	93	0.709	0.119–4.222	
	Above 40	28	1	–	
Gender	Male	168	1.751	0.885–3.466	0.142
	Female	147	1	–	
Living with family	No	231	0.813	0.396–1.669	0.573
	Yes	84	1	–	
Level of education	Diploma	16	0.823	0.140–4.817	0.829
	(Bachelor's degree)	230	0.694	0.305–1.580	
	(Master, PhD)	69	1	–	
Employment status	Governmental	250	1.397	0.609–3.201	0.43
	Private	65	1	–	
Marital status	Single	171	4.998	0.340–73.496	0.241
	Married	139	6.349	0.431–93.602	
	Divorced	5	1	–	
Salary status	10,000–15,000 SAR	168	1.153	0.473–2.809	0.754
	15,000–20,000 SAR	35	0.581	0.150–2.250	
	Above 20,000 SAR	24	1.688	0.263–10.842	
	Below 10,000 SAR	88	1	–	
Job experience	1–5 years	180	0.412	0.084–2.023	0.275
	5–10 years	69	0.689	0.200–2.375	
	Above 10 years	66	1	–	
Work position	Head	38	1.05	0.313–3.522	0.937
	Respiratory technician	28	2.956	0.655–13.334	
	Respiratory therapist 1	209	3.263	1.090–9.774	
	Supervisor	40	1	–	
Working hours per week	40–44 h	101	3.722	0.977–14.169	0.054^*^
	Above 44 h	199	2.242	0.628–8.003	
	Below 40 h	15	1	–	
Working location	Critical area	231	0.975	0.394–2.412	0.956
	Non-critical area	84	1	–	
Shift duty	Change duty (day and night)	147	1.28	0.416–3.942	0.667
	Day shift	138	1.182	0.364–3.834	
	Night shift	30	1	–	
Workload patient ratio per shift	1–10 ratio	82	0.866	0.229–3.282	0.832
	1–4 ratio	21	1.181	0.235–5.940	
	1–6 ratio	79	1.818	0.474–6.975	
	Above 1–10 ratio	76	1.332	0.376–4.715	
	(Office work)	57	1	–	

* Significant.

Results from multinomial logistic regression.

## Discussion

This cross-sectional study aimed to assess variables associated with burnout prevalence among RCPs in Saudi Arabia. The findings indicate that a significant proportion of RCPs in Saudi Arabia experienced severe burnout, aligning with earlier research conducted in Kuwait and Saudi Arabia (4, 5). This concerning trend underscores the persistent issue of burnout within the respiratory care profession, a phenomenon also reported in other countries (15–17).

Our study identified significant associations between burnout and participants aged between 31 and 40, who were more prone to severe burnout. This is consistent with previous research emphasizing age as a predictor of burnout (5). The added responsibilities and work demand experienced by individuals in this age group might contribute to this vulnerability (15, 26).

Marital status was also a significant factor, with single participants being more likely to experience severe burnout than their married counterparts. This finding aligns with previous studies (5) and may be explained by the potential lack of support systems and outlets for stress relief among single individuals (15, 18).

Furthermore, work-related factors such as longer weekly working hours and a higher patient-workload ratio were significantly associated with severe burnout. These findings highlight the necessity of managing workloads and providing adequate staffing levels to mitigate burnout among RCPs (2, 17, 26).

An intriguing finding of our study was the association between educational level and burnout. Respiratory therapists with postgraduate degrees did not appear to be more likely to experience burnout than those with diploma or undergraduate degrees. This is similar to previous research showing a negative association between educational level and burnout (4, 19). Further investigations are warranted to delve into this association and identify the underlying factors contributing to it.

Regarding burnout levels, the data reveals that there is no significant difference in burnout levels between respiratory therapists working in critical and non-critical care settings. This finding is consistent with previous research conducted in Kuwait, which also reported high levels of burnout among healthcare professionals regardless of their work environment (4, 5). It suggests that burnout is a pervasive issue affecting health care professionals including respiratory therapists across different care settings (15, 20).

The study also found a significant association between depersonalization and the number of work hours per week. Respiratory therapists who worked longer hours per week were more likely to experience severe depersonalization. This finding is consistent with previous research highlighting the relationship between long working hours and depersonalization (11). It underscores the importance of managing work hours to prevent depersonalization and potentially mitigate burnout (21).

Interestingly, the data indicates a potential association between personal achievement and job position among respiratory therapists. Supervisors appeared to have lower levels of personal achievement compared to other job positions. This observation highlights the need for further research to explore the factors contributing to personal achievement and job-specific challenges within the respiratory therapy profession in Saudi Arabia (21).

The data also suggests that there may be a trend toward lower burnout levels among respiratory therapists with more years of experience. Those with over 10 years of experience had the lowest proportion of severe burnout. This finding is consistent with previous research showing that experience may act as a protective factor against burnout (5, 22). However, the trend is not statistically significant, indicating the need for more in-depth investigation into the relationship between experience and burnout. The study also explored the association between hospital size (measured by the number of beds) and burnout levels among respiratory therapists. While there was no statistically significant difference in burnout levels based on hospital size, there was a trend suggesting that respiratory therapists in smaller hospitals may experience higher burnout levels. This observation warrants further investigation to determine whether hospital size indeed plays a role in burnout among respiratory therapists in Saudi Arabia (21, 22).

The data reveals a significant association between depersonalization and income level among respiratory therapists. Those with higher income levels were more likely to experience severe depersonalization. This finding contradicts previous research suggesting a negative association between income and burnout (4, 22). Therefore, it is essential to conduct more extensive studies to understand the unique dynamics of income and depersonalization in the context of respiratory therapy in Saudi Arabia (15, 22, 23).

## Recommendations

The implications of burnout extend far beyond individual wellbeing, encompassing the healthcare system as a whole. Burnout can adversely affect the physical and mental health of healthcare professionals, potentially compromising the quality of care provided to patients and leading to adverse outcomes (5, 25). Organizational consequences, including decreased productivity and elevated turnover rates, pose challenges to maintaining an effective healthcare workforce.

Several studies have highlighted the importance of the following strategies in addressing burnout among healthcare professionals, including respiratory therapists (15, 21, 23, 24):

1. **Workload management:** Healthcare facilities should implement workload management strategies to ensure that respiratory therapists do not face excessive work hours, which can lead to burnout and depersonalization.
2. **Support for supervisors:** Special attention should be given to supporting supervisors within the respiratory therapy profession, as they may face specific challenges related to personal achievement. Training and mentorship programs can help enhance their job satisfaction and reduce burnout.
3. **Experience recognition:** Healthcare organizations should recognize and reward the experience of respiratory therapists as it may act as a protective factor against burnout. Creating career development opportunities can help retain experienced professionals in the field.
4. **Hospital size awareness:** Smaller healthcare facilities should be aware of the potential challenges their respiratory therapists may face in terms of burnout. Additional support and resources may be required to address these issues effectively.
5. **Income-related interventions:** Organizations should consider the impact of income levels on depersonalization and explore ways to address this issue. This may include providing financial counseling and stress management programs for respiratory therapists.

## Conclusions

This study contributes significantly to the existing knowledge on burnout among RCPs in Saudi Arabia. By identifying associations between burnout and various factors, this research paves the way for the development of targeted strategies to prevent or manage burnout. It is paramount for healthcare organizations and policymakers to acknowledge the urgency of addressing burnout, as it not only affects the wellbeing of healthcare professionals but also the quality and sustainability of healthcare services.

## Limitations

While our cross-sectional study provides valuable insights, it's important to note that it cannot definitively establish causal relationships between variables. The cross-sectional design means that data was collected at a single point in time, making it challenging to determine whether changes in one variable preceded or followed changes in another. This limits our ability to definitively conclude that one variable directly causes changes in another. The study relies entirely on self-reported data, which can introduce potential biases. Respondents may exaggerate their difficulties to pressure their employers for improved working conditions, leading to overestimation of burnout prevalence. Future research endeavors should consider longitudinal designs and objective burnout measures to further investigate the factors contributing to burnout among RCPs in Saudi Arabia.

## Data Availability

The original contributions presented in the study are included in the article/supplementary material, further inquiries can be directed to the corresponding author.
