# Corrigendum: Association of burnout and working environment conditions in respiratory care professionals in Saudi Arabia: a cross-sectional study

**DOI:** 10.3389/fpubh.2025.1551184

**Published:** 2025-01-31

**Authors:** Saad Al-Anazi, Syed Shahid Habib, Thamir Al-khlaiwi, Abdulaziz Alhomaidi Alodhayani, Abdulmueen Alotaibi, Saja Aldulejan, Sufana Al Safadi, Fahad Saad Alshammari, Aqeelah Marar, Afaf Alrashdi, Alhanouf G. Almutairi, Mohammed Alshahrani

**Affiliations:** ^1^Department of Physiology, College of Medicine, King Saud University, Riyadh, Saudi Arabia; ^2^Azeer Medical Company, Riyadh, Saudi Arabia; ^3^Health Promotion and Health Education Research Chair, Medicine College, King Saud University, Riyadh, Saudi Arabia; ^4^Department of Anaesthesia Technology, College of Applied Sciences, University of Almaarefa, Dariyah, Saudi Arabia; ^5^Education Department, Respiratory Care Administration, Riyadh Second Heath Cluster, King Fahad Medical City, Riyadh, Saudi Arabia; ^6^Department of Respiratory Care, College of Applied Medical Science, Imam Abdulrahman Bin Faisal University, Damam, Saudi Arabia; ^7^Ministry of Health - Hospital Administration Affairs, Commissioning Hospital Department, Diriyah Hospital, Riyadh, Saudi Arabia; ^8^Department of Respiratory Critical Care, Respiratory Care Administration, Riyadh Second Heath Cluster, King Fahad Medical City, Riyadh, Saudi Arabia; ^9^Department of Rehabilitation Sciences, College of Health and Rehabilitation Sciences, Princess Nourah Bint Abdulrahman University, Riyadh, Saudi Arabia; ^10^Department of Respiratory Services, King Abdulaziz Medical City, Riyadh, Saudi Arabia

**Keywords:** burnout, respiratory therapists, sociodemographic factors, professional factors, personal achievement, multinomial logistic regression

In the published article, there was an error in affiliation 3, it should be “Health Promotion and Health Education Research Chair, Medicine College, King Saud University, Riyadh, Saudi Arabia”.

In the published article, there was an error in the details acknowledging King Saud University's support in the Acknowledgments statement. The correct **Acknowledgments** statement appears below.

“We would like to express our sincere gratitude to Al Maarefa University for their support, and the authors are grateful for the funding received by the Deanship of Scientific Research, King Saud University, Riyadh, the Kingdom of Saudi Arabia, through the Vice Deanship of Scientific Research Chairs.”

In the published article, the incorrect Funding statement was published. The correct Funding statement appears below.

